# Conserved Regulation of p53 Network Dosage by MicroRNA–125b Occurs through Evolving miRNA–Target Gene Pairs

**DOI:** 10.1371/journal.pgen.1002242

**Published:** 2011-09-15

**Authors:** Minh T. N. Le, Ng Shyh-Chang, Swea Ling Khaw, Lingzi Chin, Cathleen Teh, Junliang Tay, Elizabeth O'Day, Vladimir Korzh, Henry Yang, Ashish Lal, Judy Lieberman, Harvey F. Lodish, Bing Lim

**Affiliations:** 1 Stem Cell and Developmental Biology, Genome Institute of Singapore, Singapore, Singapore; 2 Immune Disease Institute and Program in Cellular and Molecular Medicine, Children's Hospital Boston, Harvard Medical School, Boston, Massachusetts, United States of America; 3 Computation and Systems Biology, Singapore–MIT Alliance, Singapore, Singapore; 4 Department of Biological Chemistry and Molecular Pharmacology, Harvard Medical School, Boston, Massachusetts, United States of America; 5 NUS Graduate School for Integrative Sciences and Engineering, Singapore, Singapore; 6 Fish Developmental Biology, Institute of Molecular and Cell Biology, Singapore, Singapore; 7 Bioinformatics Group, Singapore Immunology Network, Singapore, Singapore; 8 Genetics Branch, National Cancer Institute, National Institutes of Health, Bethesda, Maryland, United States of America; 9 Whitehead Institute for Biomedical Research, Cambridge, Massachusetts, United States of America; 10 Department of Biology, Massachusetts Institute of Technology, Cambridge, Massachusetts, United States of America; 11 Beth Israel Deaconess Medical Center, Harvard Medical School, Boston, Massachusetts, United States of America; University of California San Francisco, United States of America

## Abstract

MicroRNAs regulate networks of genes to orchestrate cellular functions. *MiR-125b*, the vertebrate homologue of the *Caenorhabditis elegans* microRNA *lin-4*, has been implicated in the regulation of neural and hematopoietic stem cell homeostasis, analogous to how *lin-4* regulates stem cells in *C. elegans*. Depending on the cell context, *miR-125b* has been proposed to regulate both apoptosis and proliferation. Because the p53 network is a central regulator of both apoptosis and proliferation, the dual roles of *miR-125b* raise the question of what genes in the p53 network might be regulated by *miR-125b*. By using a gain- and loss-of-function screen for *miR-125b* targets in humans, mice, and zebrafish and by validating these targets with the luciferase assay and a novel miRNA pull-down assay, we demonstrate that *miR-125b* directly represses 20 novel targets in the p53 network. These targets include both apoptosis regulators like *Bak1*, *Igfbp3, Itch, Puma*, *Prkra, Tp53inp1*, *Tp53*, *Zac1*, and also cell-cycle regulators like *cyclin C, Cdc25c*, *Cdkn2c, Edn1, Ppp1ca, Sel1l*, in the p53 network. We found that, although each miRNA–target pair was seldom conserved, miR-125b regulation of the p53 pathway is conserved at the network level. Our results lead us to propose that *miR-125b* buffers and fine-tunes p53 network activity by regulating the dose of both proliferative and apoptotic regulators, with implications for tissue stem cell homeostasis and oncogenesis. **

## Author Summary

MicroRNAs (miRNAs) are tiny endogenous RNAs that can regulate the expression of hundreds of genes simultaneously, thus orchestrating changes in gene networks and mediating cellular functions in both plants and animals. Although the identification of individual targets of miRNAs is of major importance, to date few studies have sought to uncover miRNA targets at the gene network level and general principles of miRNA regulation at the network level. Here we describe how *miR-125b* targets 20 apoptosis and proliferation genes in the p53 network. We found that, although each miRNA-target pair evolves rapidly across vertebrates, regulation of the p53 pathway by *miR-125b* is conserved at the network level. The structure of the *miR-125b* regulatory network suggests that *miR-125b* buffers and fine-tunes p53 network activity. This buffering feature of *miR-125b* has implications for our understanding of how *miR-125b* regulates oncogenesis and tissue stem cell homeostasis. We believe these findings on *miR-125b* support a new fundamental principle for how miRNAs regulate gene networks in general.

## Introduction

MicroRNAs (miRNAs) are short non-coding RNA molecules that were first discovered as regulators of developmental timing, and later found to regulate complex networks of genes to orchestrate cellular functions. *Lin-4* was the first miRNA gene to be discovered, and shown to regulate developmental timing by repressing its target genes at the post-transcriptional level [1]. Subsequently, miRNAs were found to regulate processes ranging from proliferation and apoptosis, to cell differentiation and signal transduction [2]–[4]. Several miRNAs are conserved in metazoan evolution, one prominent example being *lin-4* whose vertebrate homologues comprise the *miR-125a/b* family [5]. Much like *lin-4*′s role of regulating the homeostasis of reiterative or self-renewing stem cells in *C. elegans* [6], recent studies have shown that *miR-125a/b* regulates mammalian neural stem cell commitment, as well as the mammalian hematopoietic stem cell (HSC) pool size [7]–[10]. Although *Lin28* and *Bak1* have been proposed as the critical targets of *miR-125a/b* for regulating these stem cell compartments [8], [9], the hundreds of predicted targets for *miR-125a/b* suggest a more complex interplay between *miR-125a/b* and its targets in regulating proliferation and differentiation.

Depending on the cell context, *miR-125b* has been proposed to regulate both apoptosis and proliferation. *miR-125b* has been shown to downregulate apoptosis in many contexts, in some cases by repressing Tp53 and Bak1. Examples include mammalian hematopoietic stem cells, human leukemia cells, neuroblastoma cells, breast cancer and prostate cancer cells [9]–[18]. During zebrafish embryogenesis, loss of *miR-125b* leads to widespread apoptosis in a p53-dependent manner, causing severe defects in neurogenesis and somitogenesis [16]. On the other hand, *miR-125b* can also downregulate proliferation in a variety of human cancer cell-lines [19]–[23] and one of its bona fide targets Lin28, also promotes cancer cell proliferation [24]. Therefore in different contexts, *miR-125b* appears to be able to regulate both apoptosis and proliferation.

Another molecular pathway that regulates both apoptosis and proliferation is the highly conserved p53 network [25]–[28]. Due to the central role of the p53 network in these two processes, and because we found that *miR-125b* regulates both human and zebrafish Tp53 but not mouse Tp53 [16], we sought to examine if *miR-125b* regulates the p53 network in a conserved manner in vertebrates. To address this question, we used a gain- and loss-of-function screen for *miR-125b* targets in different vertebrates, and validated these targets with the luciferase assay and a novel miRNA-target pull-down assay. We demonstrate that *miR-125b* directly represses 20 novel targets in the p53 network, including both apoptosis regulators like *Bak1*, *Igfbp3, Itch, Puma*, *Prkra, Tp53inp1*, *Tp53*, *Zac1*, and also cell-cycle regulators like *cyclin C, Cdc25c*, *Cdkn2c, Edn1, Ppp1ca, Sel1l*. We found that although individual miRNA-target pairs were seldom conserved, regulation of the p53 network by *miR-125b* appears to be conserved at the network-level. This led us to propose that *miR-125b* buffers and fine-tunes p53 network dosage, with implications for the role of *miR-125b* in tissue stem cell homeostasis and oncogenesis.

## Results

### Identifying direct targets of miR-125b in the p53 network

**Figure 1:**
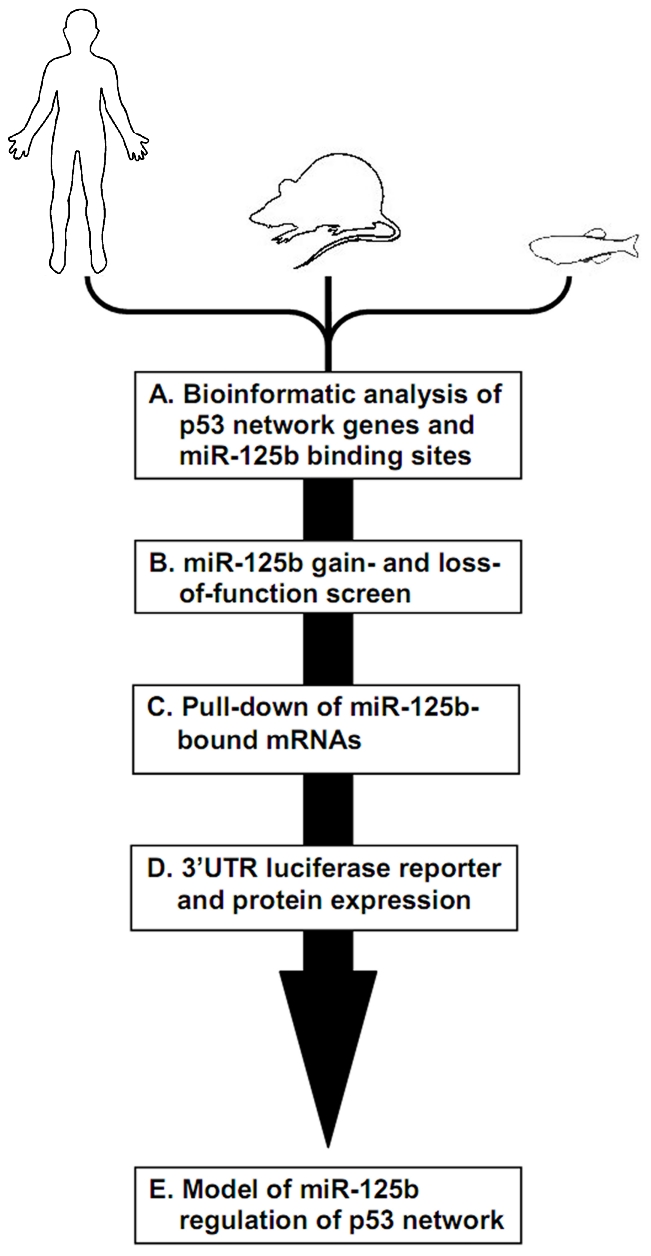
Identifying miR-125b targets in the p53 network of vertebrates. Schematic of experimental design and workflow. (A) Bioinformatic analysis was performed on p53 network genes listed in the Ingenuity Pathways Analysis database and p53 Knowledgebase, and miR-125b binding sites predicted by the TargetScan and MicroCosm databases. (B) p53 network genes were screened for miR-125b targets by using gain- (GOF) and loss-of-function (LOF) of miR-125b in human cells, mouse cells and zebrafish embryos, as indicated by effects on gene expression using qRT-PCR. (C) p53 network genes that were positive in either the GOF or LOF screen were assayed for direct binding to miR-125b using a biotinylated microRNA pull-down method. (D) p53 network genes that were also positive in the miR-125b pull-down were finally validated as miR-125b targets by 3′ UTR luciferase reporter assays and Western blots for protein expression. (E) A model of how miR-125b regulates the p53 network across vertebrates was constructed using our combined datasets for human, mouse and zebrafish cells.

To systematically identify direct targets of *miR-125b* in the p53 network of vertebrates, we first employed a bioinformatics approach by identifying all predicted *miR-125b* targets in the p53 network, followed by three complementary methods to screen and validate these targets for both direct binding and repression by *miR-125b* (Figure 1). Existing databases and prediction algorithms were used to shortlist a set of p53 network genes predicted to possess *miR-125b*-binding sites in their 3′ UTRs. We analyzed the Ingenuity Pathways Analysis™ (IPA) database and the p53 Knowledgebase [29], [30] for a list of genes and proteins that participate in the p53 network, either by regulating p53 upstream, by direct interaction with p53 protein, or by serving as effectors of p53 function downstream. We then analyzed the TargetScan and MicroCosm Target databases [31], [32] for genes that are predicted to possess *miR-125b*-binding sites in their 3′ UTRs, in three vertebrate genomes: human, mouse and zebrafish. The genes at the intersection of the predicted miR-125b target list and the list of p53 network genes constituted our list of predicted *miR-125b* targets in the p53 network (Table S1).

### miR-125b gain- and loss-of-function screen in 3 vertebrates

**Figure 2:**
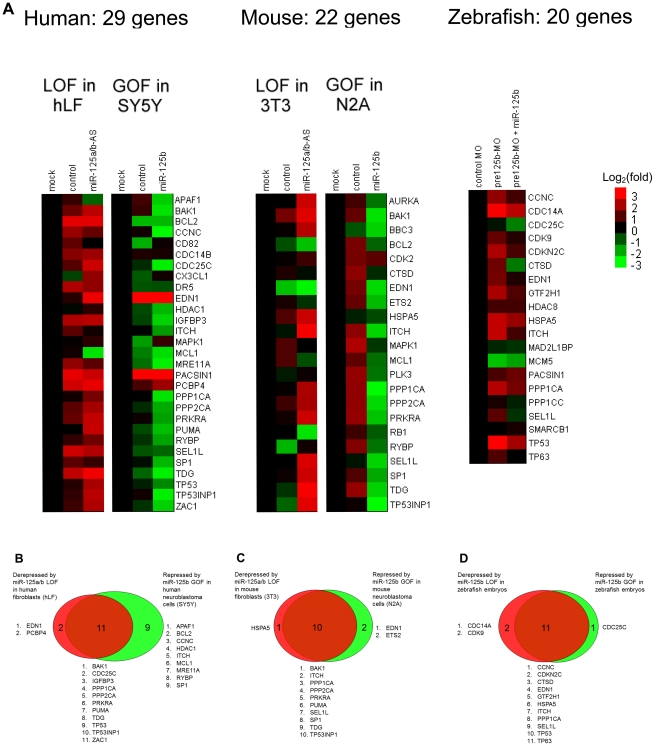
GOF/LOF screen for p53 network genes regulated by miR-125b. (A) Loss-of-function (LOF) screens were performed in human primary lung fibroblasts (hLF) or mouse 3T3 fibroblasts by transfecting an antisense RNA against both miR-125a and miR-125b (miR-125a/b-AS), or by microinjecting morpholinos (MO) against pre-*mir-125b* hairpin precursors (all 3 isoforms) into zebrafish embryos. Gain-of-function (GOF) screens were performed in human SH-SY5Y and mouse N2A neuroblastoma by transfecting the miR-125b duplex into cells in culture, or by coinjecting the miR-125b duplex with the morpholinos against pre-*mir-125b* into zebrafish embryos. Fold changes in gene expression were measured by qRT-PCR twenty-four hours after transfection or injection, relative to the mock and negative control miRNA or morpholino, and shown as log_2_(fold change) using a heat-map. (B) Human: 13 genes were significantly derepressed by a loss of miR-125b, while 20 genes were significantly repressed by a gain of miR-125b, making a total of 22 genes that passed the screen (P<0.05, fold change > 1.3, relative to mock control). (C) Mouse: 11 genes were significantly derepressed by a loss of miR-125b, while 12 genes were significantly repressed by a loss of miR-125b, making a total of 13 genes that passed the screen (P<0.05, fold change > 1.3, relative to mock control). (D) Zebrafish: 13 genes were significantly derepressed by a loss of pre-*mir-125b* (P<0.05, fold change > 1.3, relative to control MO), while 12 genes were significantly repressed/rescued by a gain of miR-125b (P<0.05, fold change > 1.3, relative to pre-*mir-125b* MO), making a total of 14 genes that passed the screen. All experiments were performed with at least three biological replicates.

Next we sought to screen our list of predicted targets for significant repression by *miR-125b* in cells, by performing a *miR-125b* gain- and loss-of-function screen. Gain-of-function (GOF) in *miR-125b* was achieved by transfection of *miR-125b* duplex into human SH-SY5Y or mouse N2A neuroblastoma cells, whereas loss-of-function (LOF) in *miR-125b* was achieved in human primary lung fibroblasts or mouse 3T3 fibroblasts by knocking down *miR-125b* with an antisense (AS) RNA (Figure 2A). We chose to perform a gain-of-function screen in human (SH-SY5Y) or mouse (N2A) neuroblastoma cells, because these cells possess low levels of endogenous *miR-125b* (Figure S1A, S1B). For the loss-of-function screen, we chose human fetal lung (hLF) or mouse (3T3) fibroblasts because they possess high levels of *miR-125b* (Figure S1C, S1D). *miR-125a*-AS was co-transfected with *miR-125b*-AS to achieve a complete silencing of the *miR-125a/b* family, because *miR-125a*, which shares the same seed sequence and the same predicted targets as *miR-125b*, is also highly expressed in human and mouse fibroblasts (Figure S1C, S1D). Genes that were either significantly repressed by *miR-125b* or significantly derepressed by *miR-125a/b*-AS with fold-changes within the range of microRNA regulation (P<0.05, fold change > 1.3), were selected as candidate *miR-125b* targets (Figure 2B–2D). For zebrafish embryos, which possess high levels of *miR-125b*, the loss-of-function (LOF) screen was performed using an antisense morpholino cocktail that blocks the loop regions of all 3 pre-*miR-125b* hairpin precursors [16]. The gain-of-function (GOF) screen was performed by co-injecting *miR-125b* duplex with the morpholino (Figure 2A). All gene expression changes were measured with at least three biological replicates using qRT-PCR.

Our GOF/LOF screen revealed that in humans, out of 29 predicted targets in the p53 network, 13 genes were derepressed by *miR-125a/b*-AS in hLF cells and 20 genes were repressed by *miR-125b* in SH-SY5Y cells (Figure 2B). In mice, out of 22 predicted targets in the p53 network, 11 genes were derepressed by *miR-125a/b*-AS in 3T3 cells and 12 genes were repressed by *miR-125b* in N2A cells (Figure 2C). In zebrafish embryos, out of 20 predicted targets in the p53 network, 13 genes were derepressed by pre-*miR-125b* morpholino and 12 genes were repressed by the injection of *miR-125b* duplex (Figure 2D). In total, 22 human genes, 13 mouse genes and 14 zebrafish genes passed the gain- and loss-of-function qRT-PCR screen.

### Direct binding interactions between miR-125b and mRNA targets from the p53 network

**Figure 3:**
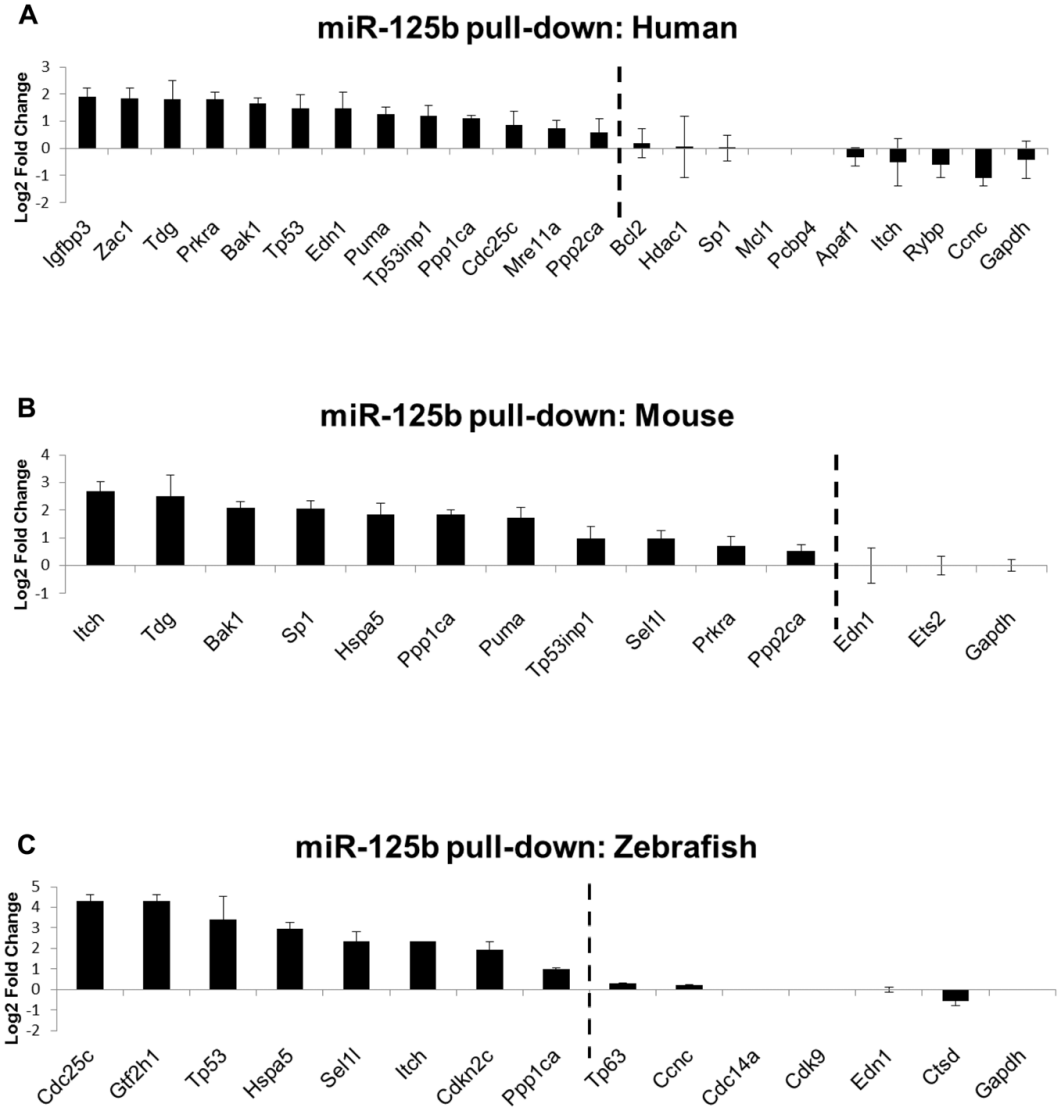
Direct binding of miR-125b to p53 network targets. Biotinylated miR-125b was used as bait to pull-down mRNAs bound to miR-125b, using streptavidin-conjugated magnetic beads. The mRNAs were quantified by qRT-PCR, normalized to *Gapdh,* and then compared relative to the same mRNA species pulled down by a biotinylated *C. elegans* negative control microRNA. The enrichment of mRNAs bound to miR-125b is presented as mean log_2_ fold change ± s.e.m. (n≥3 biological replicates). (A) Human: 13 out of 22 candidate targets were significantly enriched by miR-125b pull-down in human primary lung fibroblasts (hLF) 24 hours after transfection. (B) Mouse: 11 out of 13 candidate targets were significantly enriched by miR-125b pull-down in mouse 3T3 fibroblasts 24 hours after transfection. (C) Zebrafish: 8 of 14 candidate targets were significantly enriched by miR-125b pull-down in zebrafish embryos 24 hours after injection. Dashed line: cutoff for genes that were significantly enriched (Log_2_ Fold change > 0.5, P<0.05).

To assess which candidate *miR-125b* targets identified in the gain- and loss-of-function qRT-PCR screen are directly bound by *miR-125b* in cells, we employed a novel miRNA pull-down method developed by Lal *et al.* (manuscript in preparation). RNA transcripts bound to biotinylated-miR-125b were pulled down with streptavidin beads and quantified by qRT-PCR relative to mRNAs bound to biotinylated-control miRNA (log_2_ fold change > 0.5, P<0.05). In this assay, biotinylated miRNAs were shown to be loaded into the RNA-induced silencing complex (RISC) and fully functional in repressing their target mRNAs (Lal *et al.*, manuscript in preparation). This method provides a robust and complementary method for detecting miRNAs bound to endogenous target mRNAs, and serves as a useful approach for distinguishing direct and indirect targets in the same pathway (Lal *et al.*, manuscript in preparation). Quantification of the pulled down mRNA targets in hLF cells revealed that 13 out of 22 gene transcripts, *Bak1, Cdc25c, Edn1, Igfbp3, Mre11a, Ppp1ca, Ppp2ca, Prkra, Puma, Tdg, Tp53, Tp53inp1* and *Zac1*, were direct binding targets of *miR-125b* in human cells (Figure 3A). In mouse 3T3 cells, 11 out of 13 gene transcripts, *Bak1, Hspa5, Itch, Ppp1ca, Ppp2ca*, *Prkra, Puma, Sel1l, Sp1, Tdg* and *Tp53inp1*, were found to be direct binding targets of *miR-125b* (Figure 3B). In zebrafish embryos, 8 out of 14 gene transcripts, *Cdc25c, Cdkn2c, Gtf2h1, Hspa5, Itch, Ppp1ca, Sel1l,* and *Tp53*, were pulled down by *miR-125b* (Figure 3C). *Tp53* mRNA was pulled down by *miR-125b* only in human lung fibroblasts and zebrafish embryos but not in mouse fibroblasts, consistent with previously published results [16] and the Targetscan algorithmic prediction that *miR-125b* targets *Tp53* in humans and zebrafish but not in mice.

### Validation of miR-125b targets in the p53 network

**Figure 4:**
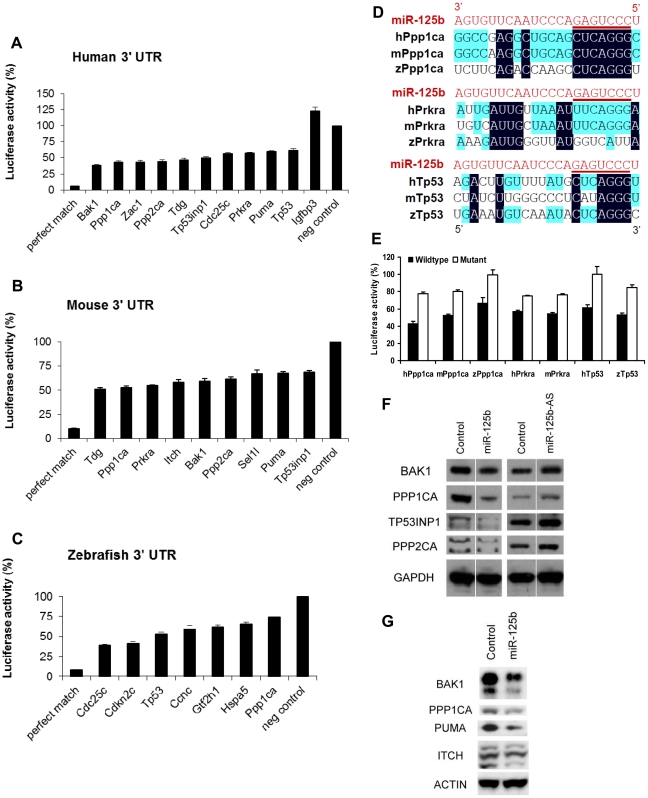
Validation of miR-125b targets. Candidate p53 network genes that were positive in both the GOF/LOF screen and miR-125b pull-down were validated for targeting by miR-125b using the 3′ UTR luciferase reporter assay and Western blots for protein expression. (A-C), Reporter genes containing the full-length 3′ UTRs of each selected target gene were co-transfected with miR-125b duplex into 293T cells. Luciferase readings were obtained 48 hours after transfection and presented here as the average percentage of luciferase activity ± s.e.m. (n≥3) relative to a scrambled duplex co-transfected control (100%). A reporter containing a 23-nucleotide-binding-site with perfect complementarity to miR-125b was used as the perfect match positive control, while the unmodified luciferase reporter was used as the empty negative control. (A) Human: 10 out of 13 candidate genes' 3′ UTRs showed significant repression by miR-125b relative to the control (p<0.01). (B) Mouse: 9 out of 11 candidate genes' 3′ UTRs showed significant repression by miR-125b relative to the control (p<0.01). (C) Zebrafish: 7 out of 8 candidate genes' 3′ UTRs showed significant repression by miR-125b relative to the control (p<0.01). (D) Alignment of predicted *miR-125b* binding sites in the 3′UTRs of Ppp1ca, Prkra and Tp53 across three species. Seed-binding sequences are underlined. Bases conserved in two (blue) or three (black) species are highlighted. (E) The 3′UTR seed-binding sequences of 7 target mRNAs were mutated and assayed for direct binding to *miR-125b* using the luciferase reporter assay, relative to wild-type 3′UTR sequences. (E) The seed-binding sequences in the 3′UTR of 7 predicted target mRNAs were mutated and compared to wild-type sequences for binding to *miR-125b* using luciferase reporter assay. (F-G) Western blot analysis of protein expression of selected target genes two days after a transfection of miR-125b duplex, miR-125b antisense (AS) or negative control duplex or negative control antisense. (F) Western blots showed that miR-125b repressed BAK1, PPP1CA, TP53, and PPP2CA levels in human SH-SY5Y neuroblastoma cells, while the antisense RNA miR-125b-AS derepressed expression of these proteins in human ReNcell VM neural progenitor cells. (G) Western blots showed that miR-125b repressed BAK1, PPP1CA, PUMA, and ITCH levels in mouse N2A neuroblastoma cells. Abbreviations: h, human; m, mouse; z, zebrafish.

As a final validation of the candidate *miR-125b* targets we have identified in the p53 network, we tested our candidate target genes with the luciferase reporter assay. Where cloning was successful, we cloned the entire 3′ UTR of selected candidate target genes into a *Renilla* luciferase reporter, and assayed luciferase expression following co-transfection of *miR-125b* duplex into HEK-293T cells. Transfection of *miR-125b* significantly suppressed 40-60% (P<0.01) of the luciferase activity of many 3′ UTR reporters of the *miR-125b* targets we analyzed, relative to transfection of the negative control miRNA (Figure 4A). For humans, the 3′ UTR reporters of *Bak1, Cdc25c, Ppp1ca, Ppp2ca, Prkra, Puma, Tdg, Tp53*, *Tp53inp1,* and *Zac1* were significantly suppressed by *miR-125b*. In mice, the 3′ UTR reporters of *Bak1, Itch, Ppp1ca, Ppp2ca, Prkra, Puma, Sel1l, Tdg,* and *Tp53inp1* were significantly suppressed by *miR-125b* (Figure 4B). In zebrafish, the 3′ UTR reporters of *Ccnc, Cdc25c, Cdkn2c, Gtf2h1, Hspa5*, *Ppp1ca,* and *Tp53* were significantly suppressed by *miR-125b* (Figure 4C). With the exception of zebrafish *Ccnc*, all genes tested were positive in the *miR-125b-*pull-down as well as the *miR-125b* gain- and loss-of-function screen. Amongst these targets, we found *Ppp1ca, Prkra* and *Tp53* to be especially interesting from the evolutionary viewpoint, since all 3 vertebrate species possess these 3 genes, but each gene shows a different pattern of evolutionary conservation with respect to *miR-125b-*repression. *Ppp1ca* is repressed by *miR-125b* in all 3 species, *Prkra* is repressed by *miR-125b* in humans and mice, while *Tp53* is repressed in humans and zebrafish. To examine the sequence evolution of these miRNA-mRNA pairs in greater detail, we compared the Targetscan-predicted *miR-125b* binding sites of these genes in humans, mice and zebrafish. In *Ppp1ca*, the predicted binding site is 95% identical between humans and mice and 55% identical between humans and zebrafish, while the seed binding sequence is 100% conserved in all 3 species (Figure 4D). In *Prkra*, the predicted binding site is 94% identical between humans and mice, but only 26% identical between humans and zebrafish, while the seed binding sequence is completely absent in zebrafish (Figure 4D). In contrast, the predicted binding site in *Tp53* is 64% identical between humans and zebrafish, and the seed binding sequence is 100% conserved between humans and zebrafish, but only 36% identical between humans and mice, while the mouse seed binding sequence has acquired 2 point mutations (Figure 4D). The *miR-125b-*repression patterns we observed for each of these genes in the qPCR, pull-down and luciferase assays are consistent with these DNA sequence analyses, suggesting that evolution in the miRNA-mRNA binding is driving the evolution in *miR-125b-*repression patterns. Introduction of point mutations into the predicted seed binding sequences abrogated *miR-125b-*repression of each target 3′UTR luciferase reporter (P<0.05), validating the predicted *miR-125b* binding sites and confirming the miRNA-mRNA sequence evolution patterns we observed (Figure 4E).

Finally, we checked *miR-125b* regulation of protein expression in a subset of p53 network targets for which reliable Western blotting was possible. *miR-125b* significantly downregulated the protein levels of human BAK1, PPP1CA, TP53INP1, PPP2CA, CDC25C, and TP53 in SH-SY5Y neuroblastoma cells (Figure 4F). In mouse N2A neuroblastoma cells, *miR-125b* significantly downregulated mouse BAK1, PPP1CA, PUMA, and ITCH protein (Figure 4G).

### miR-125b regulation of the p53 network, but not individual miRNA-target pairs, is conserved

**Figure 5:**
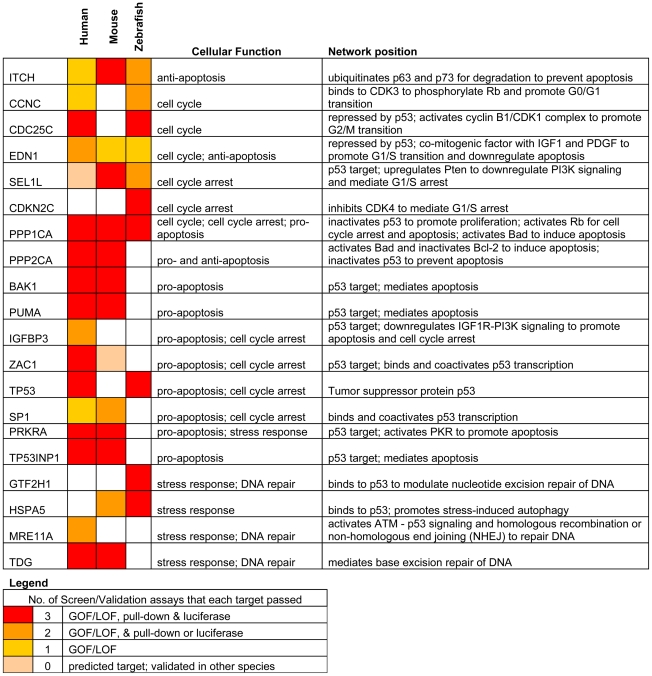
Summary of genes in p53 network that are directly targeted by miR-125b. Only targets that passed ≥ 2 validation assays, in at least one species, are shown. Red: predicted targets validated by 3 assays; Orange: predicted targets validated by 2 assays; Yellow: predicted targets validated by 1 assay; Pink: predicted targets not validated by any assay, but validated by 3 assays in another species.

**Figure 6:**
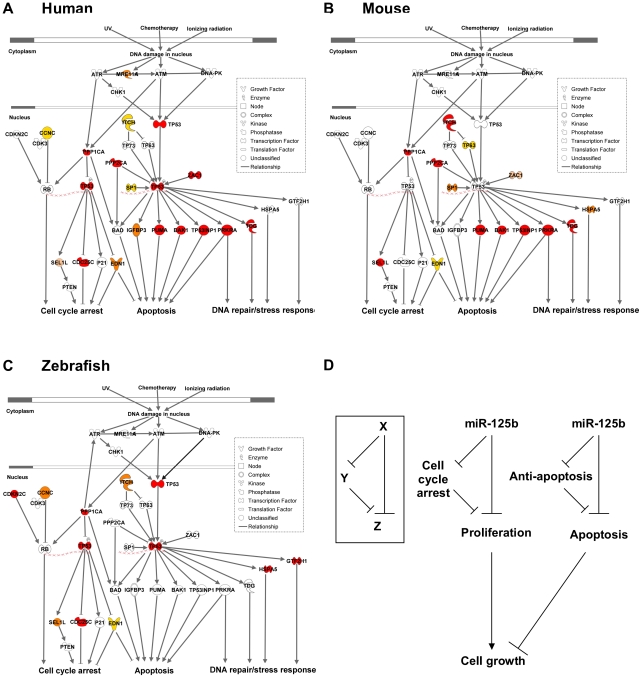
Models of miR-125b regulation of p53 networks in humans, mice, and zebrafish. (A) Human p53 network. (B) Mouse p53 network. (C) Zebrafish p53 network. Models were constructed by Ingenuity Pathway Analysis. Red: predicted targets validated by 3 assays; Orange: predicted targets validated by 2 assays; Yellow: predicted targets validated by 1 assay; Pink: predicted targets not validated by any assay, but validated by 3 assays in another species. (D) Incoherent feedforward loop (FFL) motifs characterize miR-125b regulation of p53 network genes that mediate apoptosis or cell cycle arrest.

Our results reveal that *miR-125b* regulation of the p53 network is conserved at the network-level over the course of vertebrate evolution, but individual miRNA-target pairs are evolving rapidly. To summarize our results, our list of predicted *miR-125b* targets in the p53 network (Table S1) was filtered and reclassified according to the results of the screen and validation assays (Figure 5). From the GOF/LOF screen we were able to identify mRNAs perturbed by miR-125b. However these results did not discriminate between direct or indirect targets. To supplement these experiments the pull-down assay was used to uncover mRNAs physically associated with *miR-125b*. Of note, the pull-down might not identify mRNA targets that are rapidly degraded, and as such the luciferase reporter assay can complement its shortcomings. Taken together the three assays provide a powerful means to identify direct *miR-125b* targets. In order to minimize false positives, we counted the number of assays for which each gene target was positive, and gene targets that failed to pass at least 2 assays in at least one vertebrate species were filtered out. Predicted targets that passed 3 assays (red), 2 assays (orange), 1 assay (yellow), or predicted targets that failed all assays but whose orthologues in other species passed 3 assays of direct regulation by *miR-125b* (pink), were colored as indicated (Figure 5). Using our conservative estimate of *miR-125b* targets in the p53 network, we found that in all three vertebrates we examined – humans, mice and zebrafish *– miR-125b* regulates multiple p53 network genes. This shows that *miR-125b* regulation of the p53 network is conserved at least at the network level. However very few individual gene targets of *miR-125b* in the p53 network were conserved across all three vertebrates (Figure 5; Figure 6A-6C). Instead, conserved *miR-125b* regulation of the p53 network appears to occur through evolving miRNA-target pairs in the three vertebrates – zebrafish (Figure 6A), mouse (Figure 6B), and humans (Figure 6C). In general, we observe *miR-125b* regulating 2 general classes of genes in the p53 network: (i) apoptosis regulators like *Bak1*, *Igfbp3, Itch, Puma*, *Prkra, Tp53inp1*, *Tp53*, and *Zac1*, and (ii) cell-cycle regulators like *cyclin C, Cdc25c*, *Cdkn2c, Edn1, Ppp1ca,* and *Sel1l*.

Because *miR-125b* represses both pro-apoptosis and anti-apoptosis genes, as well as both proliferation and cell-cycle arrest genes in all three vertebrates (Figure 5), *miR-125b* appears to modulate the p53 network on the whole through an incoherent feedforward loop (FFL) [33], [34] acting on the cellular processes of apoptosis and cell proliferation (Figure 6D). An incoherent type-2 FFL is a regulatory pattern in which X represses a target Z and also represses Y, another repressor of Z (Figure 6D). Incoherent FFLs have been found in the transcription factor networks of human embryonic stem cells and hematopoietic stem cells, and have been shown to modulate E2F1 dosage in the Myc-E2F1 pathway [35]-[37]. Besides accelerating responses and acting as amplitude filters [38]-[40], the incoherent FFL motif is also a noise buffering motif that reduces the variance of network dosage [41]–[43]. Thus our finding that incoherent FFLs fit the overall structure of network relationships between *miR-125b* and the p53-mediated processes, suggests that *miR-125b* is fine-tuning and buffering p53 network dosage.

## Discussion

In this study, we sought to identify direct targets of *miR-125b* in the p53 network of humans, mice and zebrafish, to better understand how *miR-125b* regulates the p53 network throughout evolution and how that might relate to its conserved role in regulating tissue stem cells.

We identified 20 direct targets of *miR-125b* in the p53 network, including 15 novel targets like *Zac1, Puma, Itch* and *Cdc25c*, and also targets like *Bak1* and *Tp53* that were identified in previous studies [9], [16]–[18]. In general, we found that *miR-125b* directly represses 2 classes of genes: apoptosis regulators and cell-cycle regulators. With the exception of *Ppp1ca, Itch* and *Edn1*, very few individual targets were strictly conserved throughout vertebrate evolution. Instead, we found that only the network-level of regulation was conserved, and *miR-125b*-regulation of individual apoptosis and proliferation regulators appears to be evolving rapidly from species to species. This observation suggests that, at least within the vertebrates, the 3′ UTR sequences of each gene target is evolving rapidly via neutral genetic drift. In other words, the loss or gain of a single *miR-125b*-binding site in the 3′ UTR of most genes appears to have a relatively insignificant effect on the fitness of an organism. On the other hand, the strict conservation of *miR-125b*-regulation at the network-level in humans, mice and zebrafish, suggests that natural selection acts on the network-level rather than the gene-level with regard to miRNA-target evolution. It will be interesting to see if this novel paradigm applies to other microRNAs or gene networks as well.

Previous studies on miRNA evolution have suggested that a relatively poor conservation of individual miRNA-target pairs but strong conservation of a miRNA-gene network relationship is consistent with miRNAs' role as buffers of gene expression [42], [44], [45]. Our observation that an incoherent FFL-like network motif fits the overall structure of the *miR-125b* - p53 network models with respect to apoptosis and cell proliferation, lends further support to this idea since incoherent FFL network motifs are well-adapted for noise filtering [41], [43], [46]. It is thought that miRNAs are at least partially responsible for the phenomenon of developmental or phenotypic stability within each species [41], [42], termed “canalization” by C. H. Waddington [47]. These studies suggest that miRNAs have a conserved role in regulating the overall stability of pathways/networks, a role which is relatively unaffected by the loss or gain of individual miRNA-targets over the course of evolution. A network buffering function has also been suggested for the regulation of muscle development by *miR-1* throughout evolution, regulation of the Wnt pathway by *miR-8,* and fine-regulation of Pten dosage by a variety of miRNAs [48]–[50]. Our findings suggest that the fine-tuning of p53 network dosage by *miR-125b* is another example of this paradigm.

Fine-regulation of p53 network dosage by *miR-125b* may also explain *miR-125b*'s conserved role in regulating tissue stem cell homeostasis. In *C. elegans*, loss-of-function mutations in *lin-4* lead to a delay in differentiation and thus expansion of vulval precursor cells, seam stem cells in the lateral hypodermis and mesoblasts, causing multiple defects in larval development [6]. In zebrafish, loss of *miR-125b* leads to widespread p53-dependent apoptosis with consequent defects in early embryogenesis, especially in neurogenesis and somitogenesis [16]. Overexpression of *miR-125a/b* causes an expansion of mammalian hematopoietic stem cells (HSCs) and aberrant differentiation, leading to myeloid leukemia [9], [10] and also lymphoid leukemia if *miR-125b* is overexpressed in fetal liver HSC-enriched cells [12]. However, the molecular underpinnings of *miR-125a/b*'s regulation of tissue stem cell homeostasis had remained unclear largely due to the complex nature of microRNA regulation of gene networks. The 2 classes of *miR-125b* targets in the p53 network, and the incoherent FFL network motifs that we found, may at least partially explain how *miR-125b* regulates tissue stem cells in vertebrates. By fine-tuning both apoptosis regulators and cell-cycle regulators, *miR-125b* may fine-tune the p53 network dosage to drive the self-renewal of tissue stem cells. It could explain how overexpression of *miR-125b* leads to an expansion of self-renewing hematopoietic stem cells while loss of *miR-125b* leads to aberrant apoptosis and proliferation, with consequent defects in tissue differentiation.

Several studies have implicated *miR-125b* as an oncogene in a variety of mammalian tissue compartments, e.g. leukemia, neuroblastoma, prostate cancer and breast cancer [9]–[18]. These studies have ascribed *miR-125b*'s anti-apoptotic effect as an oncogene to its direct suppression of Bak1 or Tp53 [9], [16]-[18]. On the other hand, several research groups have also reported *miR-125b*'s role as a potential tumor suppressor by suppressing proliferation in cell-culture models [19]–[23]. Our identification of 20 direct targets of *miR-125b* in the p53 network reconciles these findings because *miR-125b* modulates the expression of both apoptosis regulators and cell-cycle regulators. Although *miR-125b*'s suppression of p53 itself is not conserved in mice, *miR-125b*'s anti-apoptotic role – through suppression of multiple pro-apoptosis regulators in the p53 network – appears to be conserved in vertebrates. *miR-125b*'s ability to fine-tune the subtle balance of apoptosis vs. cell-cycle regulators and thus buffer the p53 network dosage in different contexts, could explain why *miR-125b* dysregulation can lead to either tumor suppression or oncogenesis depending on the context. It is possible that this buffering feature of *miR-125b* represents a general principle of miRNA regulation of gene networks.

## Materials and Methods

### Prediction of miR-125b targets in the p53 network

A list of p53-associated genes was compiled from the p53 Knowledgebase website [30] and from the Ingenuity Pathway Analysis™ database [29]. The targets of miR-125b in human and mouse were predicted by TargetScan [31]. The targets of miR-125b in zebrafish were predicted by MicroCosm [32]. The human homologues of mouse and zebrafish targets were identified by the DAVID gene ID conversion tool.

### Cell culture and transfection

Human lung fibroblast cells, human neuroblastoma SH-SY5Y cells, mouse neuroblastoma Neuro-2A cells, mouse fibroblast Swiss-3T3 cells and human HEK-293T cells were maintained in DMEM media, supplemented with 10% fetal bovine serum and 1% penicillin-streptomycin (Invitrogen). Neuro-2A cells, 3T3 cells, SH-SY5Y cells and human lung fibroblast cells were transfected in suspension with 5×10^5^ cells per well in 6-well plates using lipofectamin-2000 (Invitrogen). miRNA duplexes and antisense oligonucleotides (Ambion) were transfected at a final concentration of 80 nM.

### Microinjection in zebrafish embryos

Wild-type zebrafish were maintained by standard protocols [51]. All injections were carried out at 1–4 cell stage with 2 nl of solution into each embryo. In the knockdown experiments, miR-125b morpholinos were injected at 0.75 pmole/embryo (lp125bMO1/2/3 indicates the co-injection of three lp125bMOs, 0.25 pmole each); miR-125b duplex was injected as 37.5 fmole/embryo.

### Quantitative RT-PCR

RNA was extracted from cells or zebrafish embryos using Trizol reagent (Invitrogen) and subsequently column-purified with RNeasy kits (Qiagen). For qRT-PCR of miR-125a, miR-125b and RNU6B, 100 ng of total RNA was reverse-transcribed and subjected to Taqman microRNA assay (Applied Biosystems). For qRT-PCR of mRNAs, cDNA synthesis was performed with 1 µg of total RNA using the High Capacity cDNA Archive Kit (Applied Biosystems). The expression of all genes was analyzed by SYBR assay using the Applied Biosystems real-time PCR system or the Fluidigm 48x48 dynamic array system (Fluidigm) following the manufacturer's protocol.

### miRNA–target pull-down assay

50 ul of streptavidin coated magnetic beads (Invitrogen) were blocked with 1 mg/ml yeast tRNA and 1 mg/ml BSA in 1 ml lysis buffer (20 mM Tris pH 7.5, 100 mM KCl, 5 mM MgCl2, 0.3% NP-40) for 2 hours at 4°C and wash twice with lysis buffer. hsa-miR-125b or cel-miR-67 (negative control) duplex was synthesized with a biotin conjugated at the 3′ end of the active strand by Dharmacon Research Inc. The miRNAs were transfected into human lung fibroblasts or mouse 3T3 fibroblasts at a final concentration of 80 nM as described above. The miRNAs were also injected into zebrafish embryos at 1 to 4-cell stage at a final concentration of 37.5 fmole/embryo. After 24 hours, cells from 3 wells of fibroblasts or 50 zebrafish embryos were incubated with 500 ul cold lysis buffer containing freshly added 100 units/ml RNase inhibitor (Invitrogen) and protease inhibitor cocktail (Roche) for 20 minutes on ice. After the cell debris is removed by centrifugation, the lysate was incubated with pre-blocked streptavidin coated beads for 2 hours at 4°C. Subsequently, the beads were washed 5 times with cold lysis buffer and incubated with Trizol for RNA extraction.

### Luciferase reporter assay

The whole 3′ UTR of target genes were cloned into the psiCHECK-2 vector (Promega), between the XhoI and NotI site, immediately 3’ downstream of the *Renilla* luciferase gene. For selected targets, we introduced 3 point mutations into the 7-nt seed-binding sequence using inverse PCR with non-overlapping primers carrying the mutated sequences. 10 ng of each psiCHECK-2 construct was co-transfected with 10 nM miRNA duplexes or into HEK-293T cells in a 96-well plate using lipofectamin-2000 (Invitrogen). After 48 hours, the cell extract was obtained; Firefly and *Renilla* luciferase activities were measured with the Dual-Luciferase reporter system (Promega) according to the manufacturer's instructions.

### Western blot assay

Cells were lysed in RIPA buffer (Pierce). Proteins were separated by a 10% polyacrylamide gel and transferred to a methanol-activated PVDF membrane (GE Healthcare). The membrane was blocked for one hour in PBST containing 7.5% milk and subsequently probed with primary antibodies (Santa Cruz) overnight at 4°C. After 1-hour incubation with goat-anti-mouse HRP-conjugated secondary antibody (Santa Cruz), the protein level was detected with luminol reagent (Santa Cruz).

### Statistical analysis

Two-tail T-tests were used to determine the significance of differences between the treated samples and the controls where values were obtained from luciferase reporter assay or qRT-PCR. The tests were performed using Microsoft Excel where the test type is always set to two-sample equal variance.

## Supporting Information

Figure S1 Mature miR-125b levels before and after overexpression or knockdown. (A) The level of miR-125b in human SH-SY5Y cells one day after a transfection with mock (lipofectamin2000 only), negative control duplex (NC-DP) or miR-125b duplex (125b-DP). (B) The level of miR-125b in mouse N2A cells one day after a transfection with mock, NC-DP or 125b-DP. (C) The level of miR-125a and miR-125b in human lung fibroblasts one day after a transfection with mock, negative control antisense (NC-AS) or miR-125a antisense and miR-125b antisense cotransfection (125ab-AS). (D) The level of miR-125a and miR-125b in mouse SWISS-3T3 fibroblasts one day after a transfection with mock, NC-AS or (125ab-AS). In all panels, the levels of miR-125a and miR-125b were quantified by real-time PCR, and presented as log_2_ (fold change) ± s.e.m. (n≥3) relative to the levelsof RNU6B loading control.
(TIF)

Table S1 Genes in p53 network with predicted miR-125b binding sites. ^a^ Hsa: *Homo sapiens*, humans. ^b^ Mmu: *Mus musculus*, mice. ^c^ Dre: *Danio rerio*, zebrafish. ^d^ Non-official but common gene name that is used in this paper.
(PDF)
