# Supplementary figures and images for: Conserved Regulation of p53 Network Dosage by MicroRNA–125b Occurs through Evolving miRNA–Target Gene Pairs
Source: PLoS Genet. 2011 Sep 15;7(9):e1002242. doi: 10.1371/journal.pgen.1002242 (PMC3174204; doi:10.1371/journal.pgen.1002242)

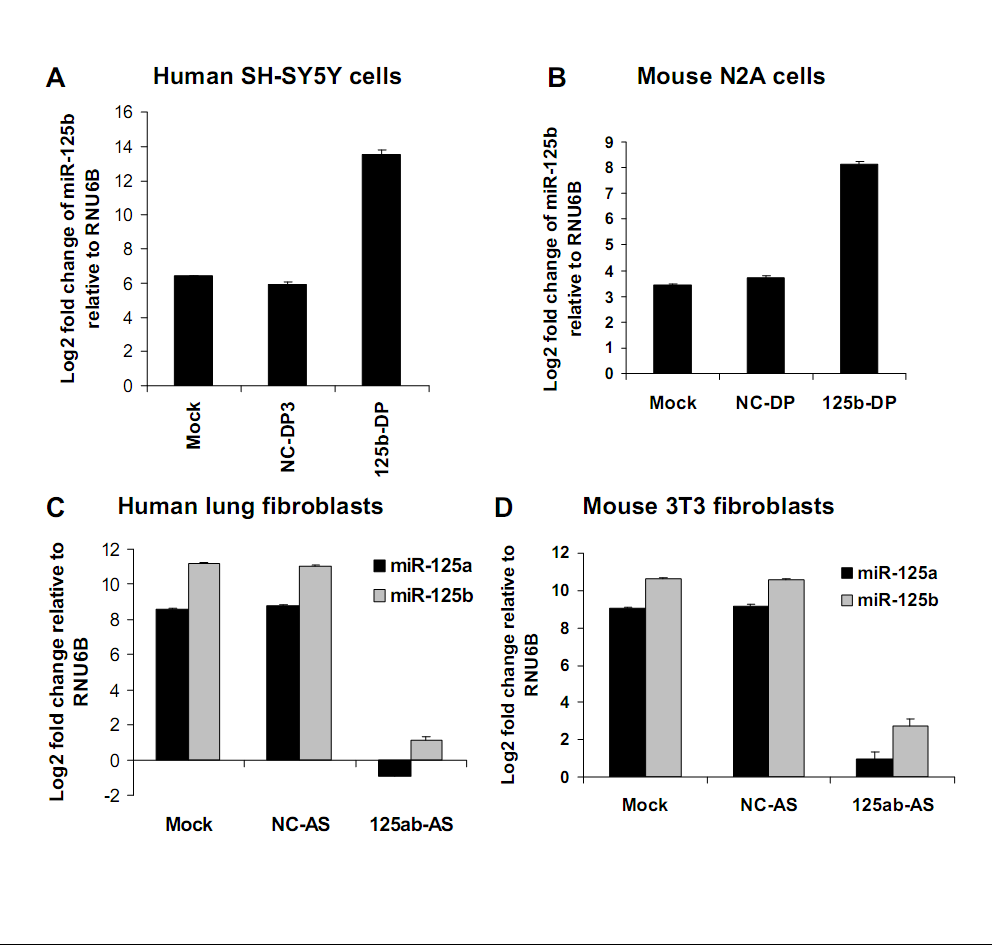

Supplement: Figure S1 — Mature miR-125b levels before and after overexpression or knockdown. (A) The level of miR-125b in human SH-SY5Y cells one day after a transfection with mock (lipofectamin2000 only), negative control duplex (NC-DP) or miR-125b duplex (125b-DP). (B) The level of miR-125b in mouse N2A cells one day after a transfection with mock, NC-DP or 125b-DP. (C) The level of miR-125a and miR-125b in human lung fibroblasts one day after a transfection with mock, negative control antisense (NC-AS) or miR-125a antisense and miR-125b antisense cotransfection (125ab-AS). (D) The level of miR-125a and miR-125b in mouse SWISS-3T3 fibroblasts one day after a transfection with mock, NC-AS or (125ab-AS). In all panels, the levels of miR-125a and miR-125b were quantified by real-time PCR, and presented as log2 (fold change) ± s.e.m. (n≥3) relative to the levelsof RNU6B loading control. (TIF) [file pgen.1002242.s001.tif]
