# Supplementary material for: Conserved Regulation of p53 Network Dosage by MicroRNA–125b Occurs through Evolving miRNA–Target Gene Pairs
Source: PLoS Genet. 2011 Sep 15;7(9):e1002242. doi: 10.1371/journal.pgen.1002242 (PMC3174204; doi:10.1371/journal.pgen.1002242)
Supplement: Table S1 — Genes in p53 network with predicted miR-125b binding sites. a Hsa: Homo sapiens, humans. b Mmu: Mus musculus, mice. c Dre: Danio rerio, zebrafish. d Non-official but common gene name that is used in this paper. (PDF) [file pgen.1002242.s002.pdf]

Supplemental Table 1. Genes in p53 network with predicted miR-125b binding sites

Genes	Description	Species	Function
1. *APAF1*	Apoptotic peptidase activating factor 1	Hsa[a]	pro-apoptosis
2. *AURKA*	Aurora kinase A	Mmu[b]	cell cycle
3. *BAK1*	BCL2-antagonist/killer 1	Hsa, Mmu	pro-apoptosis
4. *BBC3/PUMA*	Bcl-2 binding component 3; Puma[d]	Hsa, Mmu	pro-apoptosis
5. *BCL2*	Bcl2 B-cell leukemia/lymphoma 2	Hsa, Mmu	anti-apoptosis
6. *CCNC*	Cyclin C	Hsa, Dre	cell cycle
7. *CD82*	CD82 antigen	Hsa	pro-apoptosis
8. *CDC14A*	CDC14 cell division cycle 14 homolog A (S. cerevisiae)	Dre	cell cycle
9. *CDC14B*	CDC14 cell division cycle 14 homolog B (S. cerevisiae)	Hsa	cell cycle
10. *CDC25C*	Cell division cycle 25 homolog C (S. cerevisiae)	Hsa, Dre	cell cycle
11. *CDK2*	Cyclin-dependent kinase 2 (Cdk2), transcript variant 2, mRNA.	Mmu	cell cycle
12. *CDK9*	Cyclin-dependent kinase 9 (CDC2-related kinase)	Dre	transcription elongation
13. *CDKN2C*	Cdkn2c cyclin-dependent kinase inhibitor 2C; p18	Dre	cell cycle arrest
14. *CTSD*	Cathepsin D	Mmu, Dre	pro-apoptosis
15. *CX3CL1*	Chemokine (C-X3-C motif) ligand 1	Hsa	immune response; cell adhesion
16. *EDN1*	Endothelin 1	Hsa, Mmu, Dre	cell cycle; MAPK signaling
17. *ETS2*	E26 avian leukemia oncogene 2, 3' domain	Mmu	pro-apoptosis
18. *GTF2H1*	General transcription factor II H, polypeptide 1	Dre	stress response; DNA repair
19. *HDAC1*	Histone deacetylase 1	Hsa	anti-apoptosis
20. *HDAC8*	Histone deacetylase 8	Dre	anti-apoptosis
21. *HSPA5*	Heat shock 70kD protein 5 (glucose-regulated protein)	Mmu, Dre	stress response
22. *IGFBP3*	Insulin-like growth factor binding protein 3	Hsa	pro-apoptosis
23. *ITCH*	Itchy, E3 ubiquitin protein ligase	Hsa, Mmu, Dre	anti-apoptosis
24. *MAD2L1BP*	Mitotic arrest deficient 2-like 1 binding protein	Dre	cell cycle
25. *MAPK1*	Mitogen activated protein kinase 1 (Mapk1)	Hsa, Mmu	cell cycle; MAPK signaling
26. *MCL1*	Myeloid cell leukemia sequence 1	Hsa, Mmu	pro-apoptosis
27. *MCM5*	Minichromosome maintenance deficient 5, cell division cycle 46 (S. cerevisiae)	Dre	cell cycle; DNA replication
28. *MRE11A*	Meiotic recombination 11 homolog A (S. cerevisiae)	Hsa	stress response; DNA repair
29. *PACSIN1*	Protein kinase C and casein kinase substrate in neurons 1	Hsa, Mmu, Dre	endocytosis; signal transduction
30. *PCBP4*	Poly(rC) binding protein 4	Hsa	pro-apoptosis; cell cycle arrest
31. *PLAGL1/ZAC1*	Pleiomorphic adenoma gene-like 1; Zac1[d]	Hsa, Mmu	pro-apoptosis; cell cycle arrest
32. *PLK3*	Polo-like kinase 3 (Drosophila)	Hsa, Mmu	pro-apoptosis; cell

			cycle arrest
33. PPP1CA	Protein phosphatase 1, catalytic subunit, alpha isoform; PP1A	Hsa, Mmu, Dre	cell cycle; apoptosis
34. PPP1CC	Protein phosphatase 1, catalytic subunit, gamma isoform	Dre	cell cycle; apoptosis
35. PPP2CA	Protein phosphatase 2 (formerly 2A); PP2A	Hsa, Mmu	cell cycle; apoptosis
36. PRKRA	Protein kinase, interferon inducible double stranded RNA dependent activator	Hsa, Mmu	pro-apoptosis; stress response
37. RB1	Retinoblastoma 1	Mmu	cell cycle
38. RYBP	RING1 and YY1 binding protein	Hsa, Mmu	pro-apoptosis; cell cycle arrest
39. SEL1L	Sel1 (suppressor of lin-12) 1 homolog (C. elegans) (Sel1h), transcript variant 1, mRNA.	Hsa, Mmu, Dre	cell cycle
40. SMARCB1	SWI/SNF related, matrix associated, actin dependent regulator of chromatin, subfamily b, member 1	Dre	pro-apoptosis; chromatin remodeling
41. SP1	Trans-acting transcription factor 1	Hsa, Mmu	pro-apoptosis; cell cycle arrest; transcription factor
42. TDG	Thymine DNA glycosylase (Tdg), transcript variant 1, mRNA.	Hsa, Mmu	DNA repair
43. TNFRSF10B/DR5	Tumor necrosis factor receptor superfamily, member 10b; cytotoxic TRAIL receptor-2; Killer; Death receptor 5; Dr5[d]	Hsa	pro-apoptosis
44. TP53	Transformation related protein 53; p53[d]	Hsa, Dre	pro-apoptosis; cell cycle arrest; stress response
45. TP53INP1	Transformation related protein 53 inducible nuclear protein 1	Hsa, Mmu	pro-apoptosis; stress response
46. TP63	Transformation related protein 63; p63[d]	Dre	pro-apoptosis; cell cycle; differentiation

[a] Hsa: *Homo sapiens*, humans
[b] Mmu: *Mus musculus*, mice
[c] Dre: *Danio rerio*, zebrafish
[d] Non-official common gene name that is used in this paper.
